# A Novel Derivative of the Natural Product Danshensu Suppresses Inflammatory Responses to Alleviate Caerulein-Induced Acute Pancreatitis

**DOI:** 10.3389/fimmu.2018.02513

**Published:** 2018-10-30

**Authors:** Zhengnan Ren, Hongli Li, Miaoying Zhang, Yalei Zhao, Xin Fang, Xiu Li, Wei Chen, Hao Zhang, Yang Wang, Li-Long Pan, Jia Sun

**Affiliations:** ^1^State Key Laboratory of Food Science and Technology, Jiangnan University, Wuxi, China; ^2^School of Food Science and Technology, Jiangnan University, Wuxi, China; ^3^School of Medicine, Jiangnan University, Wuxi, China; ^4^Department of Pediatric Endocrinology and Inherited Metabolic Diseases, Children's Hospital of Fudan University, Shanghai, China; ^5^Department of Medicinal Chemistry, School of Pharmacy, Fudan University, Shanghai, China

**Keywords:** acute pancreatitis, danshensu-cysteine derivative, inflammation, caerulein, nutritional intervention

## Abstract

Acute pancreatitis (AP), a common abdominal inflammatory disorder, is characterized by premature intracellular activation of digestive proteases within pancreatic acini and a consecutive systemic inflammatory response. Although the mechanism remains to be fully understood, inflammation is the main cause of pancreatic damage in AP. A novel compound [4-(2-acetoxy-3-((R)-3-(benzylthio)-1-methoxy-1-oxopropan-2-ylamino)-3-oxopropyl)-1,2-phenylene diacetate (DSC)], derived from danshensu, exhibits anti-inflammatory and anti-apoptotic properties *in vitro*. However, its potential beneficial effect in AP has not been demonstrated. This study aimed to investigate the effects and underlying mechanisms of DSC in experimental AP in mice. We found that DSC suppressed inflammatory responses in AP by inhibiting the activation of nuclear factor-κB (NF-κB), signal transducer and activator of transcription 3 (STAT3) and nucleotide-binding domain leucine-rich repeat containing family, pyrin domain-containing 3 (NLRP3) inflammasome. Furthermore, treatment with DSC modulated the infiltration of neutrophils and the phenotypes of macrophages in mice induced with AP. Interestingly, we found that the expression of nuclear factor-erythroid 2 related factor 2 (Nrf2) and its regulated antioxidant enzyme heme oxygenase-1 (HO-1), which modulate inflammatory activities, was significantly increased in DSC-treated groups. Together, our findings demonstrate that DSC alleviates pancreatic inflammation and damage in AP by inhibiting the activation of NF-κB, STAT3, and NLRP3 inflammasome and modulating immune cell responses.

## Introduction

Acute pancreatitis (AP) is common and of increasing incidence in many countries (1). The majority of cases suffer from a mild form of the disease but nearly 20–30% of patients develop severe pancreatitis, associated with systemic inflammation and multiple organ dysfunction syndrome including lungs, gut, liver, and kidneys (2). AP is caused by the inappropriate activation of pancreatic enzymes by triggering an intra-acinar cascade of events, including trypsin activation (3). Although the pathogenesis of AP remains to be fully understood, data from experimental models strongly imply the key roles of inflammatory mediators and immune cell infiltration (4). Activated enzymes and inflammatory cytokines from damaged cells or infiltrated immune cells lead to tissue damage and edema (5). For example, activated neutrophils and macrophages release enzymes and cytokines such as tumor necrosis factor-α (TNF-α), interleukin-1β (IL-1β), and IL-6 (6). Recent findings indicate that activation of the nucleotide-binding domain leucine-rich repeat containing family, pyrin domain-containing 3 (NLRP3) inflammasome and nuclear factor-κB (NF-κB) pathway are involved in the development of AP (7). IL-6 is an important proinflammatory cytokine strongly linked to AP. IL-6 production correlates with the severity of disease in mice, like in the human pancreatitis (8). The expression of IL-6 promotes phosphorylation of several down-stream targets including the signal transducer and activator of transcription 3 (STAT3) (9). Meanwhile, in pancreas, the phosphorylation of STAT3 via IL-6 trans-signaling connects the pancreatic damage to systemic complications (10). Despite significant recent advances, AP is still a dangerous disease with no specific pharmacological therapy or nutritional intervention (11). Thus, to explore a novel curative strategy that inhibits inflammatory responses in AP is highly demanded.

In the progress of AP, damage-associated molecular patterns (DAMPs) are recognized by immune cell receptors including neutrophils and macrophages (12). The infiltration of neutrophils and macrophages in pancreas induce the proinflammatory reaction and activation of the inflammasome pathway (13). CD68, inducible nitric oxide synthase (iNOS) and CD206 are markers for macrophages. The iNOS is an indicator of the proinflammatory M1-phenotype and CD206 is only expressed on alternatively activated M2 macrophages.

**Figure 1:**
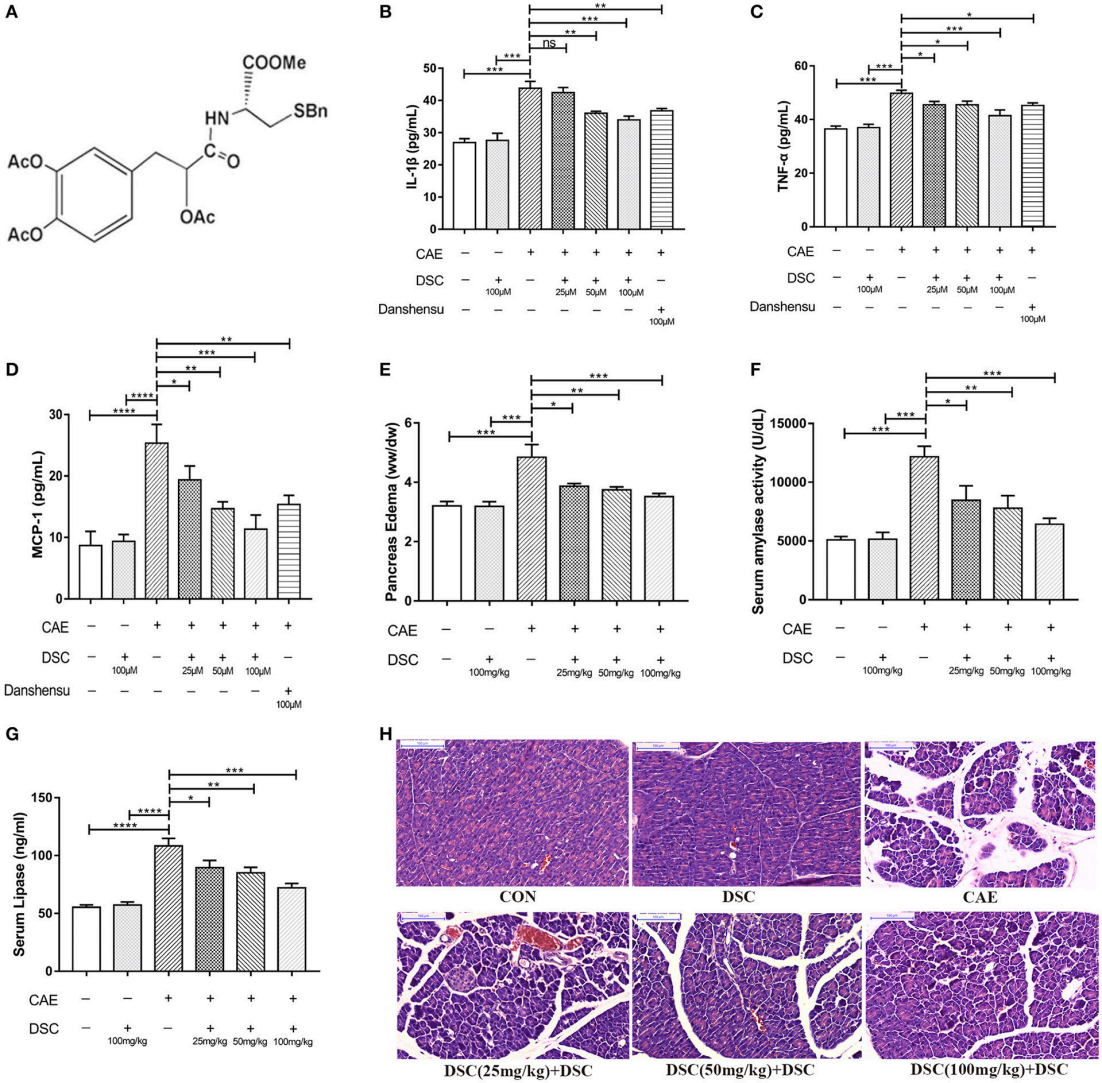
DSC suppressed caerulein-induced inflammation *in vitro* and *in vivo*. Chemical structure of DSC **(A)**, the quantitative analyzes of IL-1β, TNF-α and MCP-1 in supernatants of 266-6 cells were shown **(B–D)**. Pancreatic edema **(E)**, serum amylase **(F)**, serum lipase levels **(G)** and pancreatic histology **(H)** in BALB/c mice were determined. Control (CON): saline treatment. Caerulein (CAE): caerulein treatment. DSC, DSC-prophylactic treatment. Danshensu: danshensu-prophylactic treatment. Data shown are means ± SEM. *P < 0.05, ^**^P < 0.01, ^***^P < 0.001, and ^****^P < 0.0001.

Danshen represents one of the most versatile Chinese herbs used in food and medicine for hundreds of years (14). Danshensu, a water-soluble ingredient of Danshen, has been reported to protect against TNF-α-induced endothelial dysfunction (15) but exhibits structural instability. A novel compound [4-(2-acetoxy-3-((R)-3-(benzylthio)-1-methoxy-1-oxopropan-2-ylamino)-3-oxopropyl)-1,2-phenylene diacetate (DSC)] (Figure 1A) is synthesized by connecting danshensu and cysteine through an appropriate linkers (16). DSC has been reported to attenuate LPS-induced inflammatory responses and exhibit anti-oxidative effects *in vitro* (16, 17). However, its effects have not been demonstrated *in vivo* and associated to a specific disease. In this study, we demonstrated the potential beneficial effects of DSC on inflammatory responses associated with AP using caerulein-induced experimental models *in vitro* and *in vivo*.

## Materials and methods

### Animals and reagents

Female Balb/c mice (seven-week-old) were purchased from JOINN Laboratories (Suzhou, Jiangsu, China). and maintained in specific pathogen-free environment at the Experimental Animal Center of Jiangnan University (Wuxi, China). All mice were adjusted to laboratory conditions over the course of 1 week prior to the experiments and fasted for 12 h before induction of AP. Caerulein (CAE), dimethyl sulfoxide (DMSO), dexamethasone (DEX), and corn oil were purchased from Sigma-Aldrich Corporation (St. Louis, USA). DSC was synthesized and provided by Dr Yang Wang's laboratory in School of Pharmacy, Fudan University (Shanghai, China). The purity of the DSC was determined by high-performance liquid chromatography and the structure was identified by ^1^H NMR spectrum and ESI high resolution MS (Figures S1–S3). DSC and dexamethasone were dissolved in DMSO at 1 mg/mL and then dissolved in corn oil. The final concentration of DMSO was <1%.

### Experimental groups and animal treatment

Caerulein hyperstimulation-induced AP is the most common experimental AP model. Mice (20 ± 2g) were divided randomly into experimental groups (*n* = 7–8), as follows: saline-treated group (CON), DSC-only-treated group (DSC), caerulein-treated group (CAE), DSC-prophylactic group [DSC (prophy)], DSC-therapeutic group [DSC (thera)], and dexamethasone-treated group (DEX). All animals were given hourly intraperitoneal injection of normal saline or saline-containing caerulein (50 μg/kg) for 8 h. Corn oil-containing DSC was administered intraperitoneally at a dose of 25 mg/kg, 50 mg/kg or 100 mg/kg either 30 min before or 1 h after the first caerulein injection. Corn oil-containing DEX (25 μg/kg) was intraperitoneally infused 30 min before the first caerulein injection. For vehicle control, CON and CAE groups were given corn oil-containing DMSO (1%) by intraperitoneal injection 30 min before the first caerulein injection. One hour after the last caerulein injection, mice were sacrificed by a lethal dose of pentobarbitone. Plasma and pancreatic tissue samples were harvested for subsequent assays. All experimental procedures involving mice were carried out according to protocols approved by the Institutional Animal Ethics Committee of Jiangnan University (JN. No 20170822-20170906[107] and JN. No 20180715b0400808[148]).

### Cell culture and treatment

266–6 cells were obtained from the American Type Culture Collection (Manassas, VA, USA) and maintained in DMEM containing 1,800 mg/L NaHCO_3_, supplemented with 10% FBS, 100 U/ml penicillin, and 100 μg/ml streptomycin at 37°C in a humidified atmosphere with 5% CO_2_. Danshensu (Meilunbio, Dalian, China) and DSC were dissolved in distilled water and DMSO, respectively. The final concentration of DMSO was less than 0.1%. For treatment, cells were preincubated with DSC (25–100 μM, 4 h) or danshensu (100 μM, 4 h) before subsequent stimulation with caerulein (10 nM) for 6 h. Supernatants were then collected for cytokine measurement.

### Determination of pancreatic edema

The edema of pancreas was quantified by the ratio of wet weight to dry weight. The initial weight of the freshly harvested pancreas was defined as wet weight. The weight of the same sample after desiccation at 60°C for 72 h was served as dry weight.

### Determination of serum amylase and lipase activity

Fresh blood was collected and centrifuged at room temperature. Serum was collected and kept frozen at −80°C. Serum amylase activity was measured by an assay kit (Nanjing Jiancheng Bioengineering Institute, Nanjing, China). Lipase activity was measured by an enzyme-linked immunosorbent assay (ELISA) kit (Xinle Bioengineering Institute, Shanghai, China).

### Histopathological analysis

Fresh pancreatic samples were fixed in 4% paraformaldehyde overnight, washed with running water for 2 h, rehydrated with gradient ethanol, and then embedded in paraffin. The Skiving Machine Slicer (Leica RM2245, Wetzlar, Germany) diced 4 μm sections were stained with hematoxylin and eosin (H&E) following the standard procedure. For pancreatic morphology evaluation, a Digital slice scanner (PANNORMIC MIDI, 3DHISTCH, Hungary) was used at 200 × magnification.

### ELISA assays

IL-1β, TNF-α, IL-6, MCP-1, and IL-10 levels were measured with ELISA kits from R&D Systems (Minneapolis, MN, USA) according to the protocols of the manufacturer. Absorbance was measured at 450 nm with a microplate reader Multiclan GO (Thermos Fisher Scientific Inc, Vantaa, Finland). Data are expressed as pg/mL. Tissue samples were homogenized in a saline solution (1:19, w/v) using a Polytron homogenizer (Scientz-48, Ningbo, Zhejiang, China) at 55 Hz for 1 min. Samples were centrifuged at 4°C, 10,000 ×g for 10 min. Protein concentrations were determined by BCA Protein Assay Kit (Beyotime, Shanghai, China) during sample preparation to ensure that equal amount of total proteins were applied for cytokine measurements.

### Immunoblotting

Pancreatic samples were homogenized in ice-cold lysis buffer RIPA (containing protease inhibitors and phosphatase inhibitors). Protein concentrations were determined by using a BCA Protein Assay Kit (Beyotime, Shanghai, China). Equal amounts of protein were electrophoretic ally separated in SDS–polyacrylamide gels and then transferred onto PVDF membranes (Millipore, USA). The membranes were blocked with 5% w/v nonfat dry milk in TBS-T for 1 h at room temperature, further incubated with appropriately diluted primary antibodies overnight at 4°C and probed with secondary peroxidase-labeled antibody for 2 h at room temperature. Antibodies for p-NF-κB p65 (3033S), suppressor of cytokine signaling 3 (SOCS3; 2932T), protein kinase B (AKT; 4691S), p-AKT (4060S), extracellular regulated protein kinases 1/2 (ERK1/2; 4307S), p-ERK1/2 (4370S), NLRP3 (15101S), IL-1β (12242S), and Histone H3 (4499S) were purchased from Cell Signaling Technology (Danvers, MA, USA). Antibodies for NF-κB p65 (ab16502), STAT3 (ab68153) and p-STAT3 (ab76315) were purchases from Abcam (Cambridge, UK). The antibody for cysteinyl aspartate specific proteinase 1 (caspase-1) p20 (sc-1218) was purchased from Santa Cruz Biotechnology (CA, USA). Antibodies for erythroid 2-related factor 2 (Nrf2; 16396-1-AP) and heme oxygenase-1 (HO-1; 27282-1-AP) were purchased from Proteintech (Chicago, USA). Antibodies for GAPDH and β-actin were purchased from Bioworld Technology (Minnesota, USA). The proteins were visualized by Plus-enhanced chemiluminescence using FluorChem FC3 (ProteinSimple, USA). The densitometric analyses of protein expression by Western blot were performed by AlphaView Software (ProteinSample, CA, USA).

### Quantitative real-time PCR (qRT-PCR)

Total RNA was extracted from tissues using TRIzol following the manufacturer's protocol and cDNAs were synthesized by a reverse transcription reagent kit (TaKaRa RR036A, Japan). Gene expression levels were analyzed by qRT-PCR using the BIO-RAD CFX Connect Real-Time System (CA, USA). Primer sequences were given in Table 1. β-actin was used as a housekeeping gene.

**Table 1:** Primers used for qRT-PCR.

**Gene**	**Forward**	**Reverse**
Ly6G	5′-GACTTCCTGCAACACAACTACC-3′	5′-ACAGCATTACCAGTGATCTCAGT-3′
iNOS	5′-GTTCTCAGCCCAACAATACAAGA-3′	5′-GTGGACGGGTCGATGTCAC-3′
TNF-α	5′-GCTACGACGTGGGCTACAG-3′	5′-CCCTCACACTCAGATCATCTTCT-3′
IL-1β	5′-ATCTTTTGGGGTCCGTCAACT-3′	5′-GCAACTGTTCCTGAACTCAACT-3′
IL-6	5′-TAGTCCTTCCTACCCCAATTTCC-3′	5′-TTGGTCCTTAGCCACTCCTTC-3′
Fizz1	5′-CCAATCCAGCTAACTATCCCTCC-3′	5′-ACCCAGTAGCAGTCATCCCA-3′
CD206	5′-CTCTGTTCAGCTATTGGACGC-3′	5′-CGGAATTTCTGGGATTCAGCTTC-3′
IL-10	5′-TGGGAAGAGAAACCAGGGAGA-3′	5′-GTTTTCAGGGATGAAGCGGC-3′
Arginase-1	5′-CTCCAAGCCAAAGTCCTTAGAG-3′	5′-AGGAGCTGTCATTAGGGACATC-3′
β-Actin	5′-CCCAGGCATTGCTGACAGG-3′	5′-TGGAAGGTGGACAGTGAGGC-3′

### Immunofluorescence

4-μm–thick tissue sections were cut from paraffin-embedded blocks. Antigen retrieval was performed for 30 min. Incubation with the primary antibody was performed over night at 4°C. Secondary antibody incubation was performed for 1 h at room temperature. Anti-Ly6G (Abcam, ab25377) was used as a marker for neutrophils, anti-CD68 (Abcam, ab955) for macrophages, anti-iNOS (Abcam, ab15323) for M1 macrophages, and anti-CD206 (Abcam, ab64693) for M2 macrophages. For evaluation of immunofluorescence staining, a Digital slice scanner (PANNORMIC MIDI, 3DHISTCH, Hungary) was used at 800 × magnification.

### Flow cytometry

Freshly harvested pancreatic tissue samples were digested in 0.75 mg/mL collagenase-P (Roche Basel, Switzerland) solution at 37°C for 15 min. Subsequently, tissue was homogenized in gentleMACS™ Dissociators (Miltenyi Biotec, Bergisch Gladbach, Germany) and filtered through 75 μm filter screen with phosphate buffer saline (PBS). Single-cell suspensions were incubated for 15 min at room temperature in PBS with the following antibodies: PE/Cy7 anti-mouse CD45, Alexa Fluor 488 anti-mouse F4/80, Brilliant Violet 421 anti-mouse/human CD11b, PE anti-mouse CD206 (MMR), Alexa Fluor 647 anti-mouse Ly6G and APC anti-mouse CD86 from Biolegend (CA, USA). Isotype-matched controls were included in all experiments. Gating methods of fluorescence-activated cell sorting were programmed as CD45^+^CD11b^+^Ly6G^+^ (for neutrophils), CD45^+^CD11b^+^F4/80^+^CD86^+^ (for M1 macrophages) and CD45^+^CD11b^+^F4/80^+^CD206^+^ (for M2 macrophages). Stained cells were analyzed on an Attune NxT flow cytometer (Thermo Fisher Scientific, MA, USA).

### Statistics

Data were expressed as mean ± SEM. *P* < 0.05 was considered statistically significant. Difference among three or more groups was determined using a one-way ANOVA followed by the indicated *post hoc* test. All data were analyzed using GraphPad Prism 7 software (San Diego, CA, USA).

## Results

### DSC suppresses caerulein-induced inflammation in experimental AP and in 266-6 pancreatic acinar cells

We first studied the dose-dependent effects of DSC using 266–6 mouse pancreatic acinar cells. 266-6 cells were pretreated with ascending doses of DSC (25–100 μM) or a positive control danshensu (100 μM) for 4 h, before stimulation with caerulein (10 nM) for 6 h (16, 17). DSC, in a dose-dependent manner, suppressed caerulein-induced inflammatory responses as evidenced by reducing the production of cytokines (IL-1β, TNF-α, and MCP-1; Figures 1B–D). Notably, DSC at the same dose exhibited greater anti-inflammatory effects than danshensu (Figures 1B–D). Next, we examined varying doses of DSC in experimental AP. Mice were treated with DSC at 25, 50 and 100 mg/kg half an hour before the first caerulein injection. DSC protected pancreatic damage, which was confirmed by improved pancreatic edema, hyperamylasemia and hyperlipasemia in a dose related manner (Figures 1E–G). Histological examination of pancreatic sections confirmed an overall better index of disease severity with DSC treatment. As shown in Figure 1H, pancreatic edema, inflammatory cell infiltration and necrosis were reduced with DSC. Among the doses examined, DSC at 100 mg/kg exhibited optimal protective effects yet no cytotoxic effect and thus this dose was used for subsequent experiments.

### DSC alleviates pancreatic damage in caerulein-induced AP

**Figure 2:**
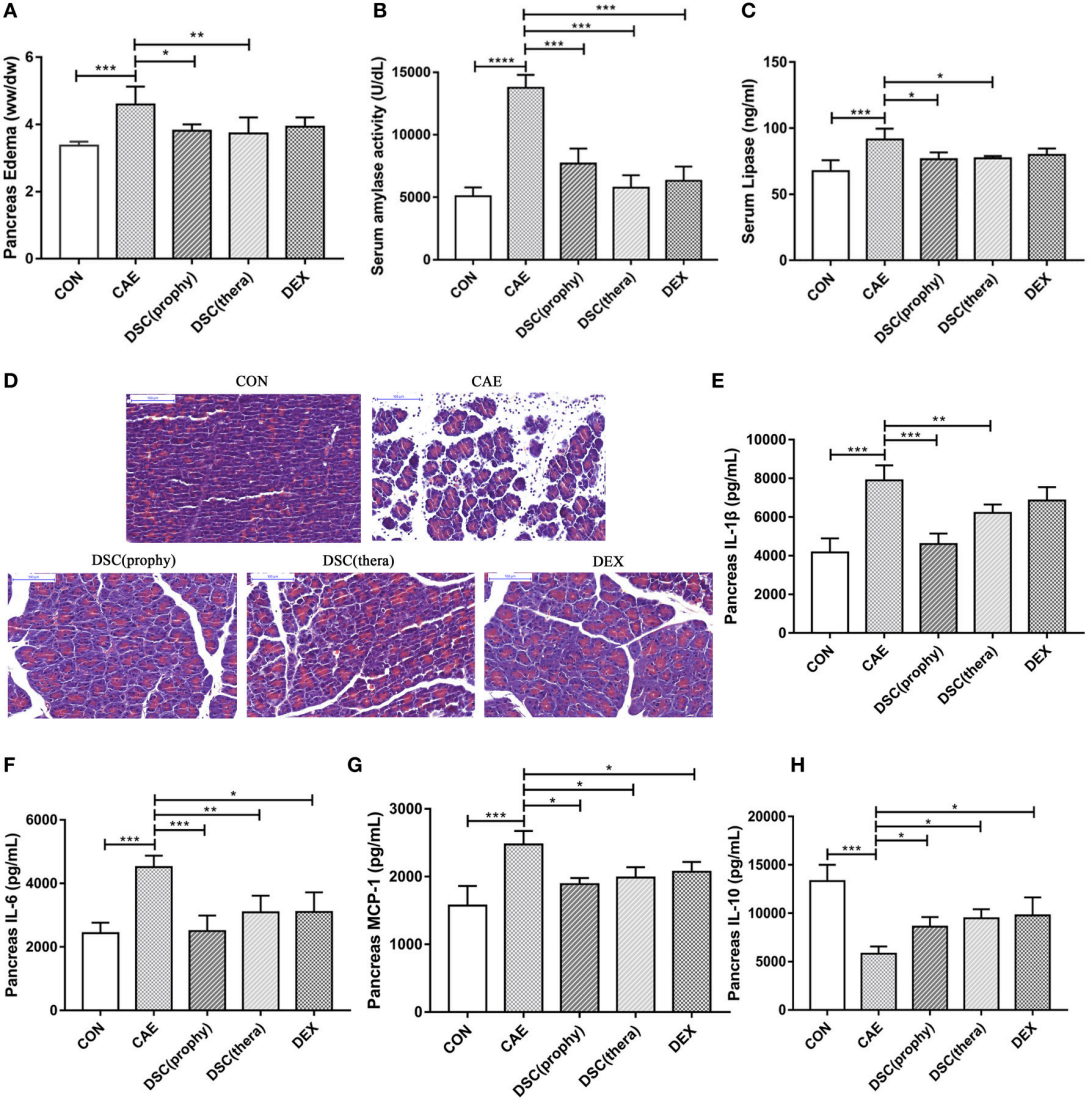
DSC alleviated the severity of AP. Pancreatic edema **(A)**, serum amylase **(B)**, serum lipase levels **(C)** pancreatic histology **(D)** and quantitative analyzes of IL-1β, IL-6, MCP-1, and IL-10 **(E–H)** were shown. Control (CON): saline treatment. Caerulein (CAE): caerulein treatment. DSC-prophylactic [DSC (prophy)]: DSC administered 30 min before starting caerulein treatment. DSC-therapeutic [DSC (thera)]: DSC administered 1 h after starting caerulein treatment. Dexamethasone (DEX): dexamethasone administered 30 min before starting caerulein treatment. Data shown are means ± SEM. ^*^P < 0.05, ^**^P < 0.01, ^***^P < 0.001 and ^****^P < 0.0001.

Mice were administered with DSC (100 mg/kg) either half an hour before (prophylactic treatment) or 1 h after (therapeutic treatment) the first caerulein injection. DEX, a synthetic adrenocortical steroid used in clinical treatment of inflammatory diseases (18), was used for positive control. The pancreatic damage was notably attenuated by both prophylactic and therapeutic treatment with DSC (Figures 2A–C). Notably, the protective effect of DSC was similar to that of DEX. The effect of DSC on AP was also confirmed by histological examination of pancreatic sections (Figure 2D). These data indicate that DSC alleviates pancreatic injury of AP in mice.

To study the effect of DSC on inflammatory responses during AP, pancreatic cytokine production was measured by ELISA. DSC reduced the production of pancreatic proinflammatory cytokines IL-1β, IL-6, and MCP-1 (Figures 2E–G). Meanwhile, the level of the regulatory cytokine IL-10 was increased in DSC-treated groups, similar to that of DEX-treated group (Figure 2H). Taken together, DSC promotes a modulatory cytokine production in pancreas in caerulein-induced experimental AP.

### DSC inhibits the activation of NF-κb, STAT3, and NLRP3 inflammasome

**Figure 3:**
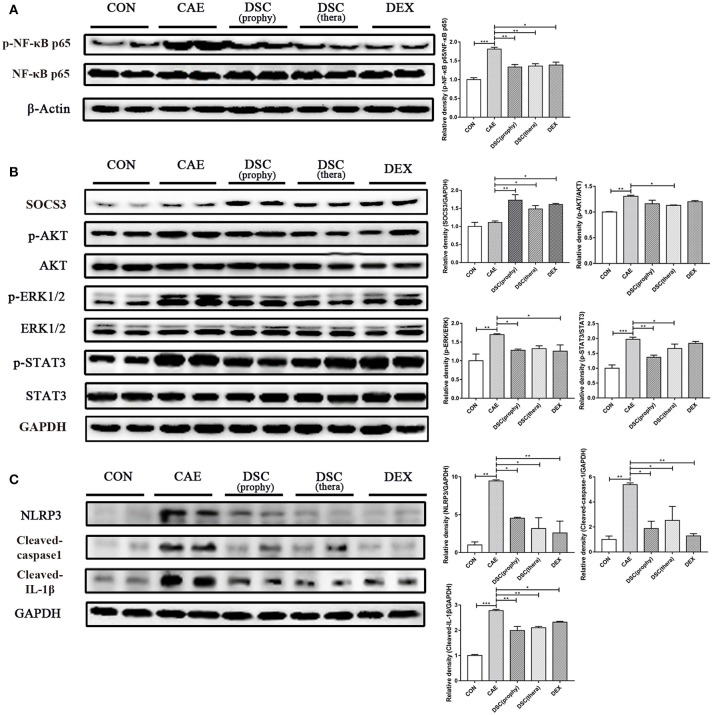
Effects of DSC on AP-mediated activation of NF-κB, STAT3, and NLRP3 inflammasome in the pancreas. The expression of pancreatic p-NF-κB p65, NF-κB p65 and β-Actin **(A)** SOCS3, p-AKT, AKT, p-ERK1/2, ERK1/2, p-STAT3, STAT3, and GAPDH **(B)** NLRP3, Cleaved-caspase-1, Cleaved-IL-1β, and GAPDH **(C)** were examined by Western blot. Data shown are means ± SEM. ^*^p < 0.05, ^**^p < 0.01, ^***^p < 0.001.

NF-κB is activated in early-phase AP and modulates multiple gene expression of proinflammatory cytokines such as TNF-α, IL-6, and IL-8 (19). Next, we measured the expression of NF-κB in pancreas by Western blot. It showed that DSC significantly attenuated AP-induced NF-κB activation (Figure 3A). The expression of IL-6 in AP was markedly reduced with DSC treatment (Figure 2F). Furthermore, the expression of SOCS3 was markedly upregulated and phosphorylation of AKT, ERK1/2, and STAT3 was significantly inhibited by DSC in pancreas during caerulein-induced AP (Figure 3B). NLRP3, an extensively investigated member of the NLR family, is responsible for the inflammasome formation and activation (20). Recently it has been shown that NLRP3 regulates the maturation and release of IL-1β in AP (4). ELISA analyses confirmed that DSC inhibited the production of the proinflammatory cytokine IL-1β (Figure 2E). The effects of DSC on the expression of NLRP3 inflammasome and its down-stream factors were examined by Western blot. As shown in Figure 3C, the activation of NLRP3, cleaved-caspase-1 and cleaved-IL-1β was alleviated by the treatment of DSC. These data suggest that DSC exerts anti-inflammatory effects on AP by partly inhibiting the activation of NF-κB, STAT3 and NLRP3 inflammasome.

### DSC ameliorates inflammation by modulating neutrophils and macrophages

**Figure 4:**
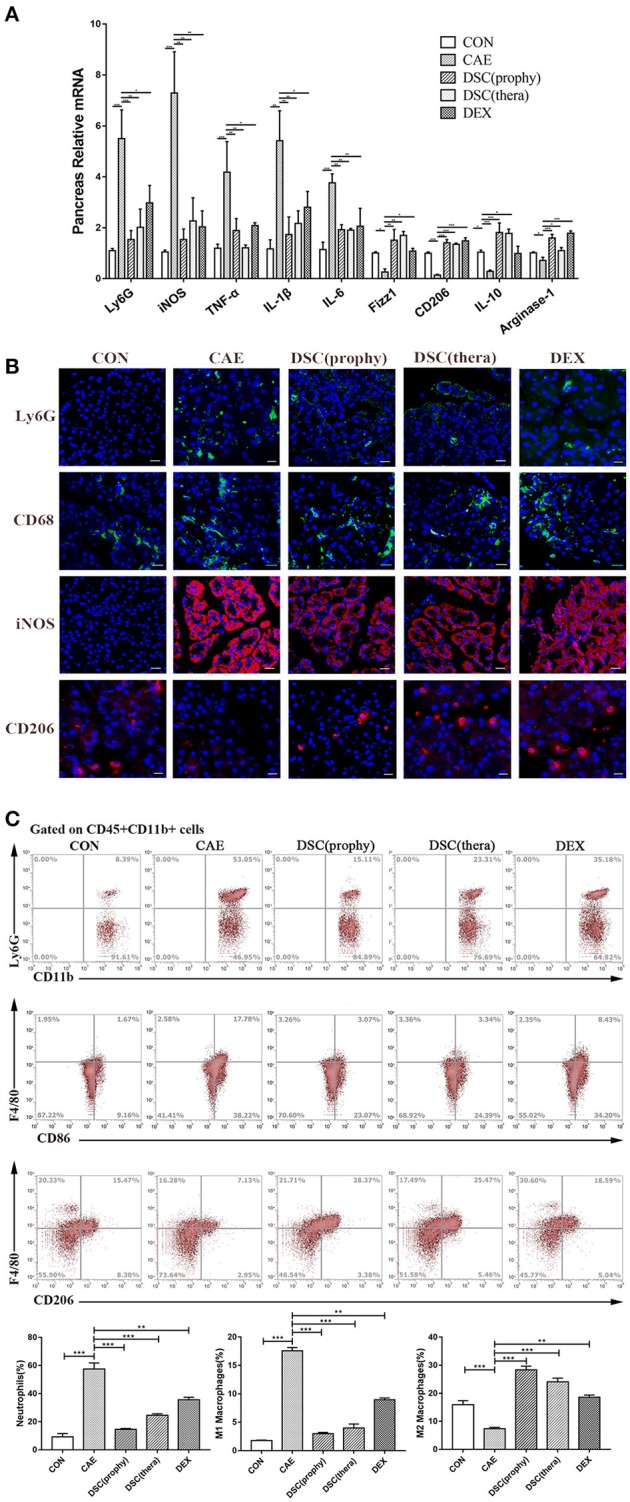
DSC modulated AP-associated immune cell responses. Relative mRNA levels of markers of immune cells in pancreas **(A)** were measured by qRT-PCR. The expression of neutrophils (Ly6G), macrophages (CD68), M1 (iNOS^+^), and M2 (CD206^+^) macrophages **(B)** were shown by immunofluorescence staining. The frequencies (gated on CD45^+^CD11b^+^ cells) of neutrophils (CD11b^+^Ly6G^+^), M1 (F4/80^+^CD86^+^), and M2 F4/80^+^CD206^+^) macrophages per pancreas **(C)** were shown by flow cytometry. Data shown are means ± SEM. ^*^p < 0.05, ^**^p < 0.01, ^***^p < 0.001.

Neutrophils and macrophages cause the release of harmful enzymes and cytokines that amplify the inflammatory responses and hence are associated with the severity of the inflammatory conditions (13, 21). Consequently, we examined the infiltration of neutrophils and M1- or M2-phenotype macrophages by qRT-PCR (Figure 4A). Ly6G was notably decreased with the treatment of DSC and DEX. The iNOS, TNF-α, IL-1β, and IL-6, markers of M1 macrophages, were suppressed by DSC. Fizz1, CD206, IL-10 and Arginase-1, markers of M2 macrophages, were markedly increased by DSC. Staining of Ly6G illustrated the infiltration of neutrophils in pancreatic tissues. Macrophages were identified by immunofluorescence stainings of CD68, Inos, and CD206. The number of infiltrating Ly6G-positive neutrophils, CD68-positive macrophages, and iNOS-positive macrophages was decreased significantly in the treatment of DSC, whereas M2 macrophages were found increased (Figure 4B). Pancreatic neutrophils (CD45^+^CD11b^+^Ly6G^+^), M1 (CD45^+^CD11b^+^F4/80^+^CD86^+^), and M2 (CD45^+^CD11b^+^F4/80^+^CD206^+^) macrophages were further quantitated by flow cytometry (Figure 4C). The percentages of neutrophils and M1 macrophages which were increased in AP, were markedly reduced in mice treated with DSC. In contrast, the percentage of pancreatic M2 macrophages was increased by DSC. These data suggest that DSC reduces the infiltration of neutrophils and phenotypic conversion of macrophages in caerulein-induced AP. Collectively, the data indicate that DSC attenuates inflammation by modulating neutrophils and macrophages.

### DSC enhances Nrf2 and Nrf2-regulated antioxidant enzyme expression

**Figure 5:**
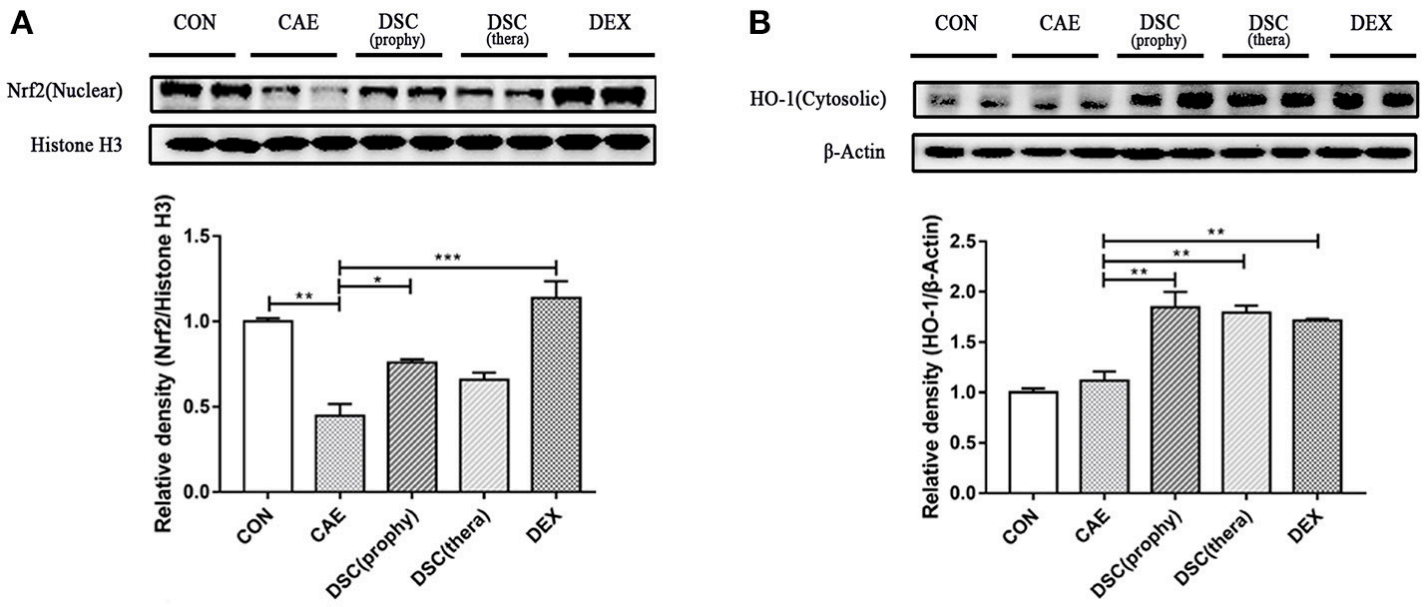
Effects of DSC on Nrf2 and HO-1 expression. The expression of nuclear Nrf2 **(A)** and cytosolic HO-1 **(B)** in caerulein-treated pancreatic acinar cells was measured by Western blot. Data are means ± SEM. Data shown are means ± SEM. ^*^p < 0.05, ^**^p < 0.01, ^***^p < 0.001.

Nrf2, a redox-sensitive transcription factor, is activated by reactive oxygen species (ROS) (22, 23) and its expression is decreased upon induction of AP in mice (4). Hence, we investigated the effects of DSC on the expression of nuclear Nrf2 and its-regulated antioxidant enzyme heme oxygenase-1 (HO-1) in caerulein-induced AP. Treatment with DSC significantly elevated the levels of Nrf2 (nuclear) and HO-1 (Figures 5A,B). These data suggest that DSC contributes to the induction of Nrf2 and Nrf2-regulated antioxidant enzyme.

## Discussion

**Figure 6:**
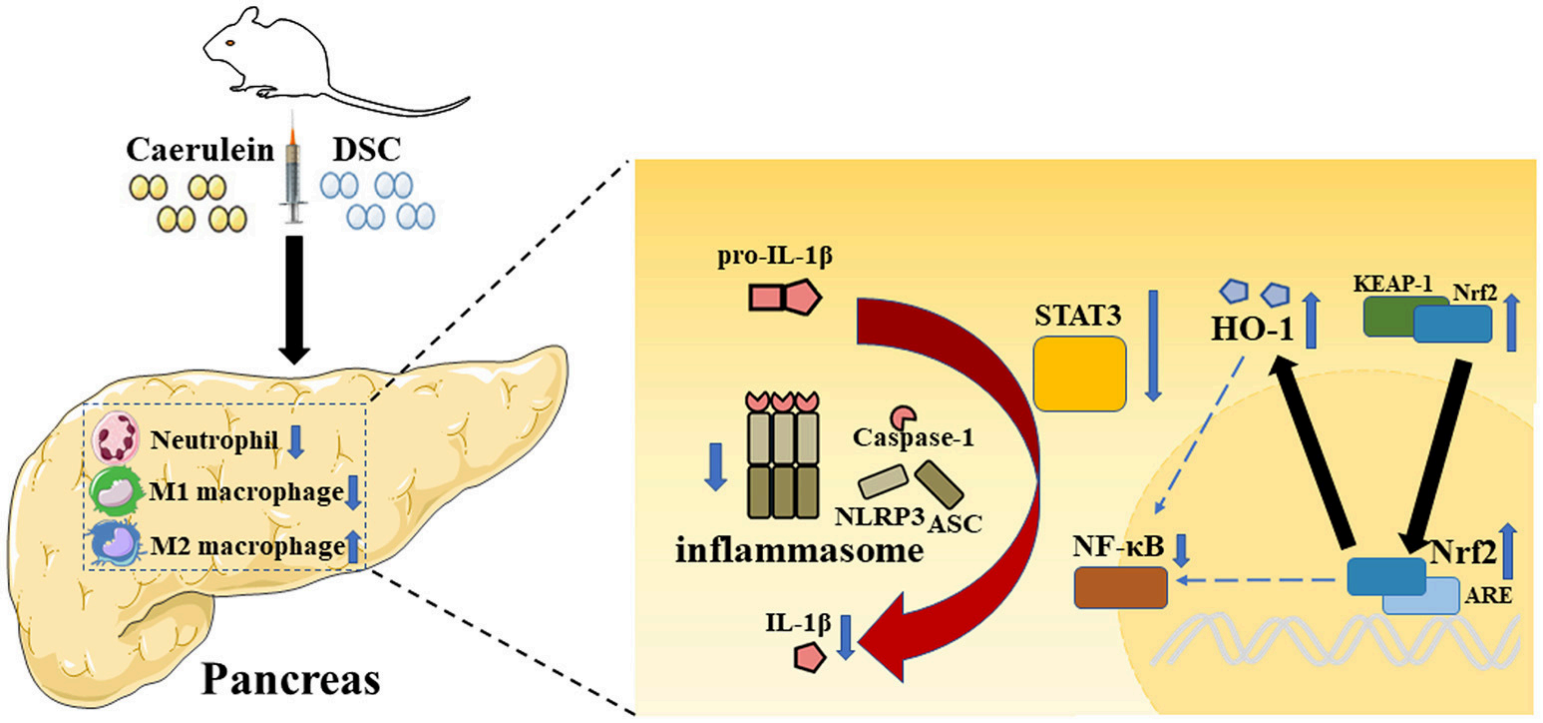
A schematic representation of modulatory effects of DSC on inflammatory pathways during AP. Caerulein induces inflammation in mice with AP. The role of DSC on AP in protecting pancreatic damage and subsequent inflammatory responses was illustrated.

The current study demonstrated protective effects of DSC on the development of experimental AP. It alleviated the severity of AP by modulating the production of inflammatory cytokines via inhibition of NF-κB, STAT3 and NLRP3 inflammasome. Importantly, DSC reduced the infiltration of neutrophils and promoted modulatory phenotypic conversion of macrophages. In addition, DSC upregulated the antioxidant enzyme HO-1 through Nrf2 activation to alleviate pancreatic damage during AP (Figure 6).

Following acinar cell injury, the vital cause for AP, damaged cells release DAMPs that activate the immune system (1). Excessive local inflammatory responses can lead to systemic inflammatory response syndrome and multi-organ dysfunction (11). Thus, strategic approaches that focus on suppressing inflammation during AP remain promising. DSC as an effective anti-inflammatory compound has not been evaluated in AP.

Earlier, by using RAW 264.7 macrophages, we demonstrated that DSC alleviated LPS-induced inflammatory responses in macrophages via suppression of phosphatidylinositol 3-kinase (PI3K)/Akt signaling pathway and NF-κB activation (17). Here, we extended our investigation in experimental AP model and our data revealed that DSC suppressed caerulein hyperstimulation induced AP in mice. Further investigation on intracellular signaling pathways demonstrated that DSC suppressed inflammatory responses by enhancing SOCS3 expression and suppressing activation of NF-κB and STAT3. NF-κB activation in acinar cells is a key intracellular event in AP and modulates a variety of inflammatory genes (24). STAT3 is activated by IL-6 trans-signaling and involved in pancreatic damage in AP (9, 10). SOCS3 functions to negatively regulate NF-κB and STAT3 signaling pathways (25). In addition, it is essential for M1 macrophage polarization and thus modulating M1 macrophage-mediated inflammatory responses (26). NLRP3 inflammasome has emerged as a vital key in the pathogenesis of AP (27). NLRP3 blockade has been shown to prevent the development of AP (27). In the absence of NLRP3, caerulein-induced pancreatic edema and inflammation are reduced (7). Our study, in agreement with previous studies, has demonstrated that DSC directly suppresses the activation of NLRP3 inflammasome in caerulein-induced AP.

It is widely held that neutrophil infiltration plays a prominent role in mediating tissue damage (28). Activated neutrophils could contribute to the inflammatory microenvironment and release inflammatory cytokines (29). Systemic depletion of neutrophils reduces pancreatic damage in caerulein-induced AP (30). Meanwhile, macrophage activation is also a hallmark of inflammation in AP (31). Macrophages, dependent on the tissue and immunological environment, may differentiate into two specific types, classical activated (M1) macrophages and alternatively activated (M2) macrophages (32). M1 macrophages are characterized by TNF-α, which enable to promote the release of proinflammatory cytokines thus exaggerating the inflammatory response in AP (1). On the contrary, M2 macrophages have the effect in restraining inflammation in pancreatitis (33). Our data showed that DSC ameliorated the severity AP by inhibiting the infiltration of neutrophils and modulating the differentiation of macrophages. Collectively, anti-inflammatory effect of DSC is exerted at least in part by inhibiting NLRP3 inflammasome and modulating immune cell responses.

In the pathogenesis of AP, activation of digestive zymogens and intra-acinar cell damage are accompanied with oxidative stress. ROS is reported to be a vital effector in AP (34). With the production of ROS in AP, antioxidant enzymes like HO-1 are secreted to protect cells against cellular oxidative damage (4). Nrf2, a member of the Cap′ N′ Collar family, is constitutively presented in the cytoplasm under steady state (35). It is degraded through Keap1-mediated ubiquitination and then comes into the nucleus (36). The activation of Nrf2 in the nucleus leads to its binding to the antioxidant response element. HO-1 is one of the main effectors of Nrf2-dependent cell antioxidant responses (37). It has been demonstrated that the activation of NF-κB and the production of proinflammatory cytokines are increased in Nrf2^−/−^ mice (38, 39). In addition, there is a close relation between HO-1 and NF-κB activation. HO-1-mediated anti-inflammatory activities suppress NF-κB signaling in severe AP (40). Here, we demonstrated that DSC promoted the expression of Nrf2 and HO-1 and inhibited NF-κB activation. These data suggest that anti-inflammatory effects of DSC in AP may be related to Nrf2 and Nrf2-regulated antioxidant enzyme.

Intriguingly, we observed comparable effects of DSC to dexamethasone in AP in terms of immuno-modulation. There have been side effects associated with clinical application of dexamethasone (41). Traditional Chinese herb and its derivatives constitute an important component for functional food with higher safety for developing preventive or therapeutic strategy.

In summary, we found that DSC, a novel compound derived from danshensu, protects against experimental AP. Our findings suggest that DSC has the potential for beneficial interventions for AP.

## Author contributions

ZR, HL, MZ, YZ, XF, and XL performed experiments and analyzed data. WC, HZ, and YW provided intellectual inputs, contributed to the data acquisition and critically reviewed the manuscript. JS, L-LP, and YW designed and interpreted experiments. JS, ZR, and L-LP wrote the paper.

### Conflict of interest statement

The authors declare that the research was conducted in the absence of any commercial or financial relationships that could be construed as a potential conflict of interest.

## Supplementary material

Figure S1 High-performance liquid chromatography of DSC.

Figure S2 ^1^H NMR spectrum of DSC (CDCl_3_, 400 MHz).

Figure S3 ESI high resolution MS date report of DSC.
