# Supplementary material for: A Novel Derivative of the Natural Product Danshensu Suppresses Inflammatory Responses to Alleviate Caerulein-Induced Acute Pancreatitis
Source: Front Immunol. 2018 Oct 30;9:2513. doi: 10.3389/fimmu.2018.02513 (PMC6218618; doi:10.3389/fimmu.2018.02513)
Supplement: Figure S1 — High-performance liquid chromatography of DSC. [file Data_Sheet_1.PDF]

Data File D:\DATA\YXC\2018-06-05 DSC-C10-05-18.D
Sample Name: DSC-C

===
Acq. Operator   : SYSTEM
Sample Operator : SYSTEM
Acq. Instrument : 1260   Location :   82
Injection Date : 6/5/2018 10:14:53 AM
                                              Inj Volume : 3.000 µl
Acq. Method   : C:\CHEM32\1\METHODS\Sample_Default_Gradient .M Last changed   : 6/4/2018 4:16:22 PM by SYSTEM
Analysis Method : D:\Methods\20170929_MeOH.M
Last changed   : 10/7/2018 2:58:43 PM by SYSTEM
                  (modified after loading)
Additional Info : Peak(s) manually integrated

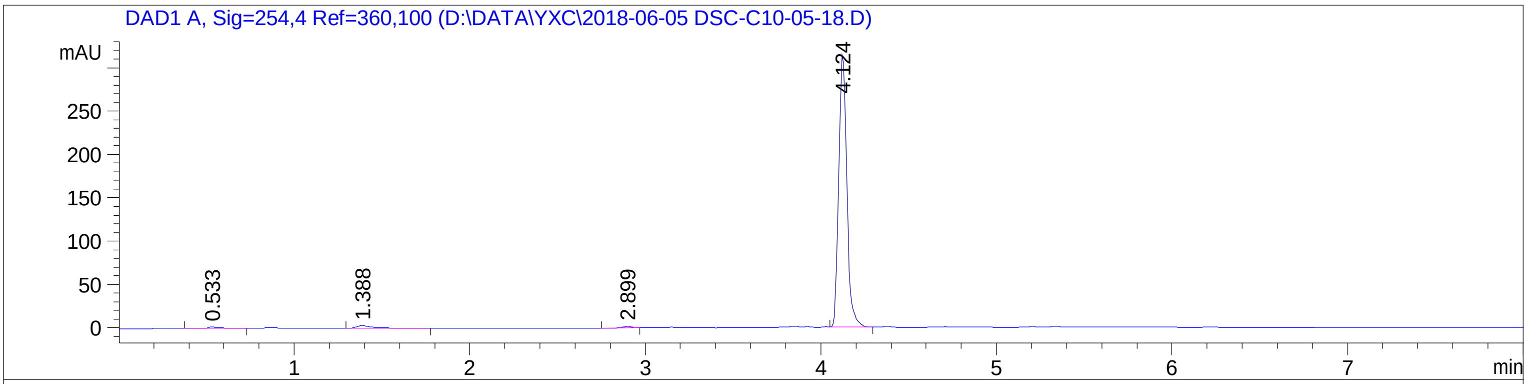

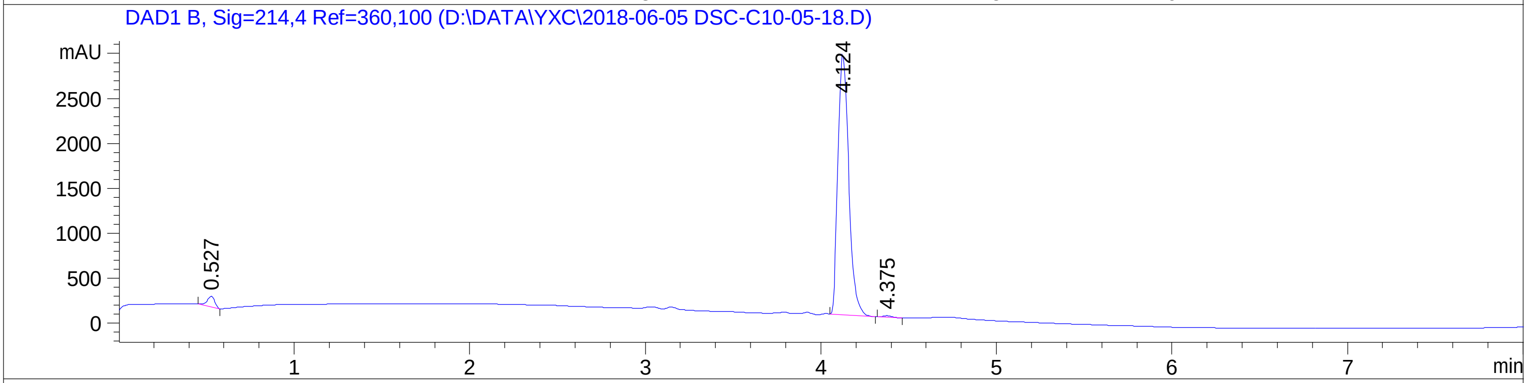

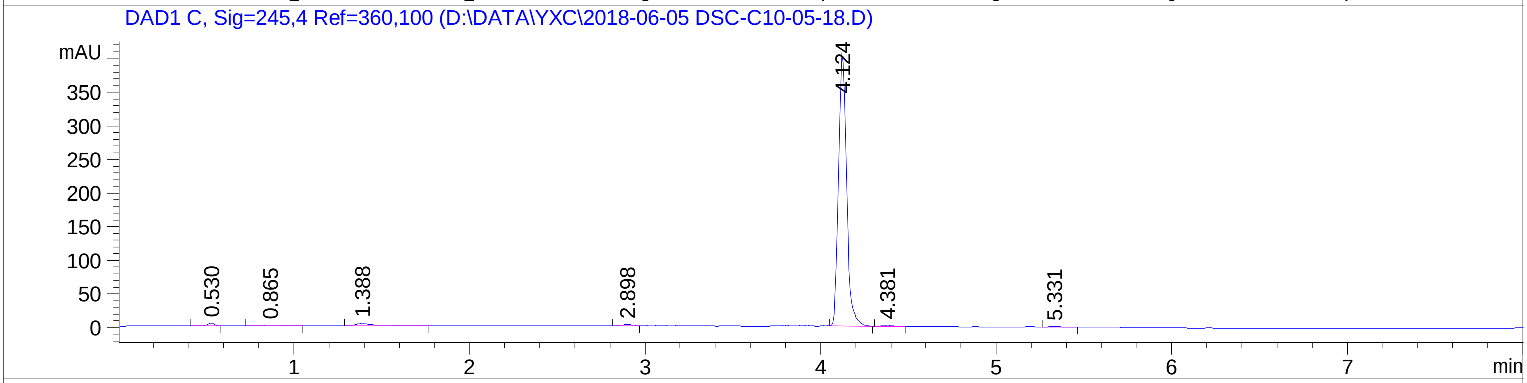

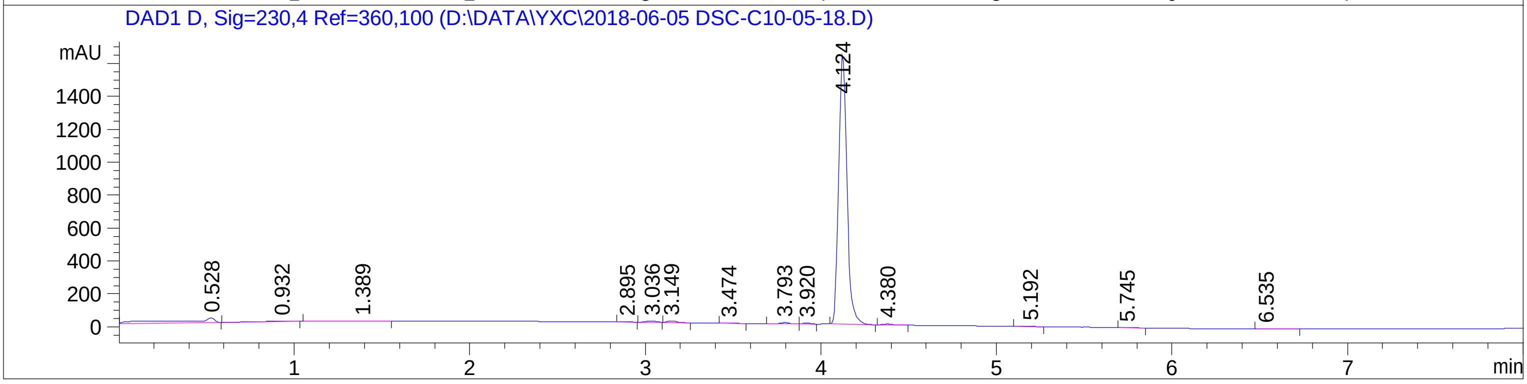

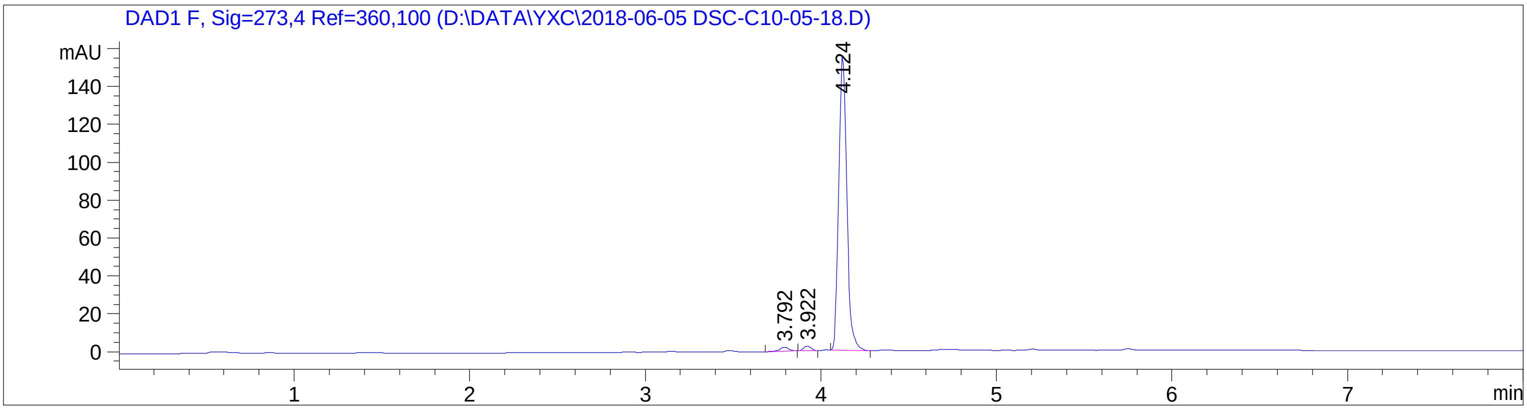

===

Area Percent Report

===

Sorted By : Signal
Multiplier : 1.0000
Dilution : 1.0000
Use Multiplier & Dilution Factor with ISTDs

Signal 1: DAD1 A, Sig=254,4 Ref=360,100

Peak #	RetTime [min]	Type	Width [min]	Area [mAU*s]	Height [mAU]	Area %
1	0.533	BB	0.0712	7.77828	1.53336	0.7434
2	1.388	BB	0.0946	20.21016	3.07645	1.9315
3	2.899	BB	0.0598	6.33697	1.68616	0.6056
4	4.124	BB	0.0515	1012.01227	314.57559	96.7195

Totals : 1046.33768 320.87156

Signal 2: DAD1 B, Sig=214,4 Ref=360,100

Peak #	RetTime [min]	Type	Width [min]	Area [mAU*s]	Height [mAU]	Area %
1	0.527	BB	0.0488	353.76932	118.57388	2.7340
2	4.124	BB	0.0702	1.25241e4	2908.57520	96.7894
3	4.375	BB	0.0532	61.66556	18.33943	0.4766

Totals : 1.29395e4 3045.48850

Signal 3: DAD1 C, Sig=245,4 Ref=360,100

Peak #	RetTime [min]	Type	Width [min]	Area [mAU*s]	Height [mAU]	Area %
1	0.530	BB	0.0458	11.52085	3.98149	0.8454
2	0.865	BB	0.1045	7.63773	1.03066	0.5604

Peak #	RetTime [min]	Type	Width [min]	Area [mAU*s]	Height [mAU]	Area %
3	1.388	BB	0.0951	21.93817	3.32094	1.6098
4	2.898	BB	0.0597	7.80902	2.08380	0.5730
5	4.124	BB	0.0516	1303.10034	403.91269	95.6203
6	4.381	BB	0.0560	5.41975	1.50725	0.3977
7	5.331	BB	0.0650	5.36037	1.22598	0.3933

Totals : 1362.78623 417.06281

Signal 4: DAD1 D, Sig=230,4 Ref=360,100

Peak #	RetTime [min]	Type	Width [min]	Area [mAU*s]	Height [mAU]	Area %
1	0.528	BB	0.1747	397.21213	28.47409	6.6296
2	0.932	BB	0.2304	40.46108	2.24403	0.6753
3	1.389	BB	0.1058	11.08576	1.47323	0.1850
4	2.895	BB	0.0560	8.61243	2.50935	0.1437
5	3.036	BB	0.0716	30.95064	7.27523	0.5166
6	3.149	BB	0.0524	32.22602	9.78857	0.5379
7	3.474	BB	0.0535	9.62067	2.83811	0.1606
8	3.793	BB	0.0547	23.60421	6.45205	0.3940
9	3.920	BB	0.0470	14.17861	5.01048	0.2366
10	4.124	BB	0.0523	5377.50879	1639.77710	89.7518
11	4.380	BB	0.0540	19.90935	5.80949	0.3323
12	5.192	BB	0.0619	10.35436	2.41970	0.1728
13	5.745	BB	0.0590	10.50049	2.60586	0.1753
14	6.535	BB	0.0633	5.30574	1.25511	0.0886

Totals : 5991.53027 1717.93241

Signal 5: DAD1 F, Sig=273,4 Ref=360,100

Peak #	RetTime [min]	Type	Width [min]	Area [mAU*s]	Height [mAU]	Area %
1	3.792	BB	0.0531	7.21101	2.04470	1.4019
2	3.922	BB	0.0484	7.66594	2.60203	1.4903
3	4.124	BB	0.0514	499.50116	155.76659	97.1078

Totals : 514.37811 160.41331

===

*** End of Report ***
