# Supplementary figures and images for: A Novel Derivative of the Natural Product Danshensu Suppresses Inflammatory Responses to Alleviate Caerulein-Induced Acute Pancreatitis
Source: Front Immunol. 2018 Oct 30;9:2513. doi: 10.3389/fimmu.2018.02513 (PMC6218618; doi:10.3389/fimmu.2018.02513)

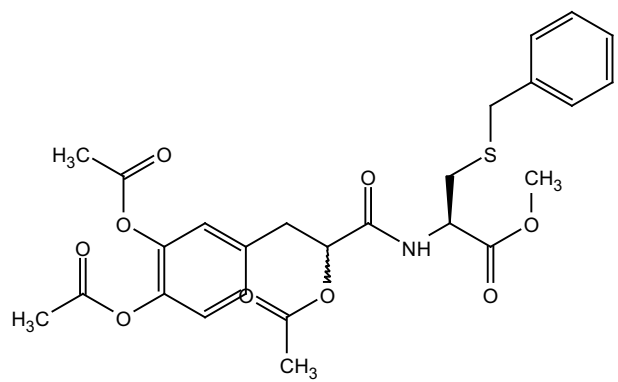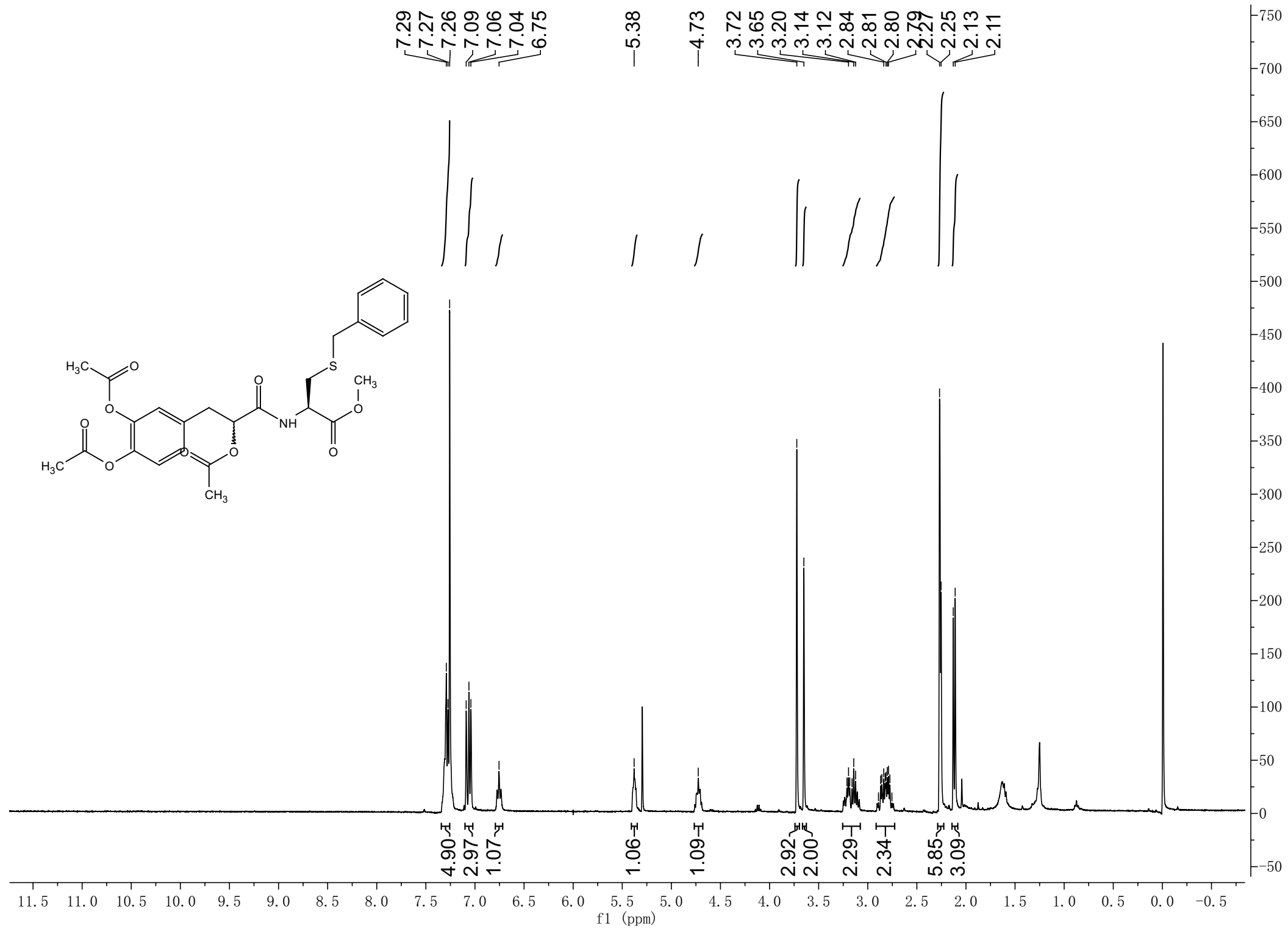

Supplement: Figure S2 — 1H NMR spectrum of DSC (CDCl3, 400 MHz). [file Data_Sheet_2.PDF]
