# Supplementary material for: A Novel Derivative of the Natural Product Danshensu Suppresses Inflammatory Responses to Alleviate Caerulein-Induced Acute Pancreatitis
Source: Front Immunol. 2018 Oct 30;9:2513. doi: 10.3389/fimmu.2018.02513 (PMC6218618; doi:10.3389/fimmu.2018.02513)
Supplement: Figure S3 — ESI high resolution MS date report of DSC. [file Data_Sheet_3.PDF]

State Key Laboratory of Organometallic Chemistry
Shanghai Institute of Organic Chemistry
Chinese Academy of Sciences
ESI High Resolution MS Date Report

Data Filename 2V67.d
Sample Name 2V67
User Name
Acquired Time 4/13/2016 10:37:18 AM
Instrument
Agilent Technologies 6224 TOF LC/MS

User Spectra

Fragmentor Voltage	Collision Energy	Ionization Mode
150	0	ESI

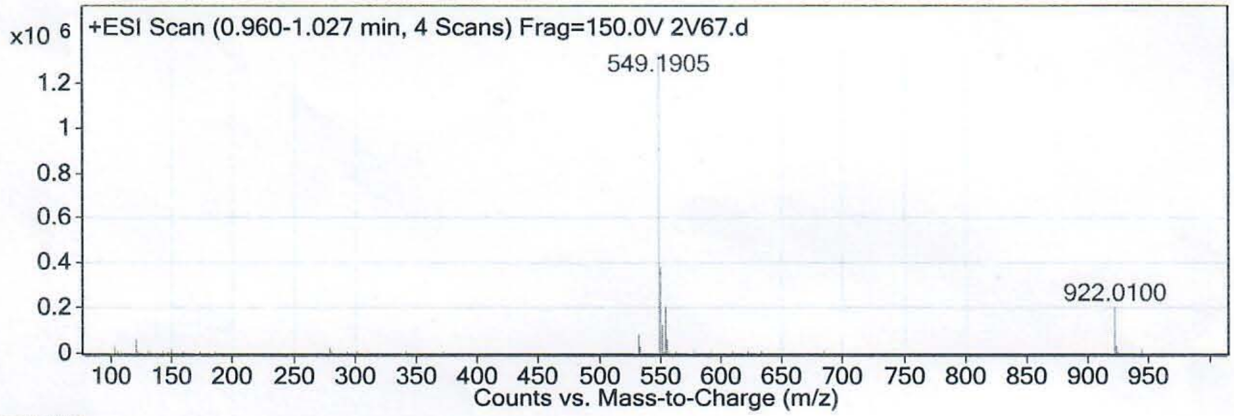

Peak List

m/z	z	Abund	Formula	Ion
532.1636		82613.2		
549.1905	1	1344354.9	C26 H33 N2 O9 S	(M+NH4)+
549.2564		70145.7		
550.1936	1	381838.9	C26 H33 N2 O9 S	(M+NH4)+
551.1922	1	121945.4	C26 H33 N2 O9 S	(M+NH4)+
554.1456		201319.2		
922.01		199178.2		

Formula Calculator Results

IonFormula	Measured Mass	Tgt Mass	Diff (ppm)	Score
C26 H33 N2 O9 S	549.1905	549.1901	-0.69	98.91
C27 H29 N6 O5 S	549.1905	549.1915	1.82	95.82
C24 H31 N5 O8 S	549.1905	549.1888	-3.23	94.45
C29 H31 N3 O6 S	549.1905	549.1928	4.36	87.38

--- End Of Report ---
